# A virtual training program for improving cultural competence among academic nurse educators

**DOI:** 10.1186/s12909-023-04414-x

**Published:** 2023-06-16

**Authors:** Monireh Rahimi, Sedigheh Khodabandeh Shahraki, Farhad Fatehi, Jamileh Farokhzadian

**Affiliations:** 1 Student Research Committee, Razi Faculty of Nursing and Midwifery, Kerman University of Medical Sciences, Kerman, Iran; 2 Department of Community Health Nursing, Razi Faculty of Nursing and Midwifery, Kerman University of Medical Sciences, Kerman, Iran; 3 School of Psychological Sciences, Monash University, Melbourne, Australia; 4 Centre for Online Health, the University of Queensland, Brisbane, Australia; 5 Nursing research center, Kerman University of medical science, Kerman, Iran

**Keywords:** Online training, Cultural care, Culturally congruent care, Culturally competent care, Cultural competency, Nursing faculty, Nursing education

## Abstract

**Background:**

Everyday, nursing students interact with culturally diverse clients. Nursing education recognizes that cultural competence is a necessary outcome of nursing programs. Nurse educators expect all nursing students to provide culturally congruent care to multicultural clients. Therefore, nurse educators must be culturally competent in order to prepare culturally competent nursing students for clinical practice. This study aimed to evaluate the effect of virtual training program on the cultural competence of academic nurse educators.

**Methods:**

This randomized controlled study included nurse educators working in six nursing schools affiliated with medical universities of Kerman province in southeastern Iran. Sixty-nine nurse educators were randomly assigned to the intervention (n = 35) and control (n = 34) groups. The training program consisted of three 2-hour sessions for a month. Cultural Diversity Questionnaire for Nurse Educators Revised (CDQNE-R) was used to evaluate the cultural competence of educators before and one month after the virtual training program.

**Results:**

Both the intervention (3.29 ± 0.58) and control (3.24 ± 0.58) groups demonstrated a similar level of cultural competence before the training program (t = 0.05, *p* = 0.95). After the training, the intervention group showed a significant increase in cultural competence (3.80 ± 0.7) compared to the control group (3.23 ± 0.67). This improvement resulted in culturally competent participants becoming culturally proficient, as evidenced by a large effect size (t = -4.76, *p* = 0.001).

**Conclusion:**

The virtual training program had a positive impact on the cultural competence of nurse educators. Given the importance of cultural competence in nursing education, continuing education programs that focus on strengthening the cultural competence of nurse educators should be prioritized. The experiences gained from implementing virtual training programs can serve as a valuable resource for nurse educators seeking to enhance their cultural competence.

## Introduction

The impact of globalization, migration, and the increasing cultural diversity among clients present a challenge to nurses’ practice and nursing education worldwide. Providing culturally congruent care to clients with diverse cultures has become a crucial aspect of healthcare service and educational programs, designed to meet the needs of families, individuals, communities, and populations from culturally diverse background. Culturally congruent care, also known as culturally competent care, is a critical component of patient-centered care and holistic care for healthcare providers in the current context of global diversity [1]. Culturally congruent care involves an understanding of how culturally based care, actions, and decisions can influence clients’ cultural values, beliefs, worldviews, and lifestyles to improve their health and well-being, or to prevent illness, disabilities, or death [2].

Cultural competence is a fundamental skill for nurses in delivering culturally congruent care [1], and it represents the process by which nurses demonstrate an understanding of and sensitivity to the cultural background of their patients a. It is a nurse’s capacity to improve the health and well-being of clients whose cultural backgrounds are different from their own [3]. Cultural competence is also essential for nursing students and nurse educators in all learning-teaching approaches [4]. Nurse educators have to teach students how to practice cultural competence in academia in order to effectively meet the needs of diverse populations in clinical settings [5]. Leininger, the founder of Culture Care Theory (CCT), emphasized the critical importance of specific cultural concepts in cultural competence education. Nurse educators must understand these concepts in order to teach culturally competent care to nursing students [6, 7]. The culture care theory emphasizes the importance of preparing nurse educators and clinicians to meet the needs of diverse populations. This approach aims to prevent cultural imposition practices, ethnocentrism, and cultural conflicts in clinical settings. A transcultural nursing curriculum based on the principles of CCT can be instrumental in developing cultural competence among nurse educators and students before graduation [2].

Iran is a multi-cultural and multi-ethnic country, with followers of different religions, including Islam, Judaism, and Christianity, and various ethnic groups such as Persians, Turks, Azari, Kurds, Gilak, Lor, Arab, Mazani, Baloch, Bakhtiari, Talash, and Turkmen. Each group has its unique identity, beliefs, and cultural values [8]. Additionally, Iran is one of the main areas of immigrants and refugees globally, particularly from Afghanistan and Iraq, who comprise a significant part of Iran’s population. In the province of Kerman, which is Iran’s largest province, cultural and ethnic diversity is high due to immigration from different races and ethnicities, primarily from Afghanistan, and occasionally banishment from neighboring provinces [9]. This diversity has led to a varied cultural profile and healthcare clients’ opinions, posing a challenge to nurses who care for them daily [10]. Nursing students are as diverse as healthcare clients, so it is crucial to continually develop the cultural competence of students and professors through education in universities. Moreover, students’ cultural diversity and behavioral characteristics can influence the cultural competence they acquire from educational centers [11].

A review of the literature shows that few studies have evaluated the cultural competence of academic nurse educators and effectiveness of educational programs in improving their cultural competence. The studies have found that many nurse educators have inadequate cultural competence, with the majority of them having a low to moderate level of cultural competence and feeling unprepared to teach cultural care. This finding suggests that nurse educators with inadequate cultural competence may struggle to teach culturally congruent care to new nurses, which in turn may hinder their ability to provide culturally competent care and to respond to issues related to cultural diversity in healthcare. The researchers strongly recommend strengthening the cultural competence of nurse educators through continuing education programs [6, 12–19]. To this end, a study examined the effectiveness of cultural care education in cultural competence of nurse educators who taught and mentored students from culturally diverse backgrounds in clinical education. The study showed a significant increase in educators’ cultural competence after the educational project. Researchers recommended workshops and enrichment projects to enhance educators’ cultural competence and mentoring skills [20]. Despite the existence of educational standards set by accrediting bodies for nursing education, little is known about the feelings of nurse educators regarding teaching cultural competence in academia. Therefore, future research could help to identify unknown factors that affect the cultural competence of nurse educators [5]. Moreover, Summers (2017) has expressed concerns about the lack of formal training and inadequate preparation of nurse educators for their teaching positions. Nurse educators should be adequately prepared for their roles as teachers, and nursing students should be prepared for their roles as graduates. To improve the quality of teaching and enhance the cultural competence of nurse educators, it may be helpful to observe the teaching methods of experienced educators and study their feedback [21].

The literature review highlighted that nurse educators’ cultural competence was a key factor in preparing students for culturally congruent care, and that transcultural nursing education was crucial to improve their cultural competence. Given Iran’s multicultural and multi-ethnic context, developing cultural competence among nurse educators is effective for the future of nursing education. However, there is a lack of research on nurse educators’ cultural competence levels and the effectiveness of continuing education and training programs in increasing their competence in different contexts and cultures. To address this gap in the literature, the present study aimed to evaluate the effect of a virtual training program on the cultural competence of nurse educators.

It sought to answer the following research question:

### Research question

What is the effect of a virtual training program on cultural competence among nurse educators?

## Methods

### Study design and settings

The present study was a randomized controlled conducted with a pretest-posttest design and intervention and control groups. The study settings included six governmental nursing schools affiliated with medical universities of Kerman province in southeastern Iran, including Kerman, Bam, Jiroft, Zarand, Sirjan, and Rafsenjan. At the time of data collection, there were approximately 1,370 nursing students enrolled in these schools, with 450 undergraduate, graduate, and doctoral students in Kerman, 140 undergraduate students in Bam and Jiroft, and 140 undergraduate students in Zarand. Sirjan had 150 nursing students, and Rafsanjan had 350 undergraduate and graduate students.

In Iran, nursing educational programs are offered at the baccalaureate, master’s, and PhD degree levels. To be admitted to governmental or Azad universities, students must hold a secondary school diploma and pass the entrance exam. Governmental universities do not charge tuition fees, whereas students at Azad University, a private institution, are required to pay necessary expenses. Additionally, the PhD degree program is only offered at governmental universities, which are supervised by the Ministry of Health and the Ministry of Sciences. The baccalaureate degree in nursing is the primary pathway to enter the nursing profession in Iran. The program typically lasts for four years and involves clinical education starting from the second term and continuing through the completion of the sixth term, in conjunction with theoretical subjects. The seventh and eighth terms are dedicated to practical training programs. Currently, nursing educational programs across Iran are standardized and developed under the supervision of the Supreme Council of the Ministry of Health. These programs include theoretical subjects, training, and internship courses offered in various educational and university-affiliated hospitals. In clinical settings, nursing students receive direct supervision and guidance from nurse educators, but in the final year, student activities are primarily performed under the supervision of nursing personnel, with alternate supervision from nurse educators. During their years of study, nursing students in Iran have the opportunity to interact with patients in the various clinical areas and gain valuable experience. Students’ progress in clinical settings starts with simple issues and gradually moves toward more complex cases. Currently, Iranian nurses must hold a bachelor’s degree from accredited universities confirmed by the Ministry of Health to work in this field. The majority of nurses in hospitals have bachelor’s degrees and are recruited using the same pattern. There are no registered nurses (RN) in the hospitals. Master’s and PhD degree programs are available for nurses who wish to become nurse educators. After obtaining a bachelor’s degree and passing the entrance exam, nurses are eligible to continue their studies in one of the following master’s degree majors: community health nursing, medical surgical nursing, neonatal intensive care nursing, critical care nursing, psychiatric nursing, geriatric nursing, and nursing management. After completing their master’s degree, graduates mainly become responsible for nurses’ education or clinical settings. The duration of the master’s program is 2.5 years, and nurses holding a master’s degree are eligible to continue their studies in a PhD program in nursing after passing the entrance exam. The PhD program typically lasts for about 4–5 years, and its graduates mainly work in educational and research positions in the field of nursing.

The cultural care competence program was introduced to the American nursing curriculum in 1966. However, in Iran, despite the first nursing school’s establishment by an American religious group, cultural issues received minimal attention in the nursing education program. Even today, cultural care remains a low priority in nursing education in Iran and is not addressed in the official program [22]. However, students still learn cultural care through a hidden program. As for nurses, they acquire cultural competence through their work experience in the healthcare environment [23].

### Population study and sample

The research population consisted of all nursing faculty members affiliated with the universities of medical sciences in Kerman province in 2022 (N = 83). The nursing faculties in Kerman were 22, 6 in Zarand, 11 in Jiroft, 5 in Sirjan, 21 in Rafsanjan, and 18 in Bam. Due to the limited number of the research population, all faculty members were invited to participate in the study by census.

Finally, sixty-nine nurse educators volunteered to participate in the study and were allocated to the intervention (n = 35) and control (n = 34) groups using a table of random numbers.

**Fig. 1:**
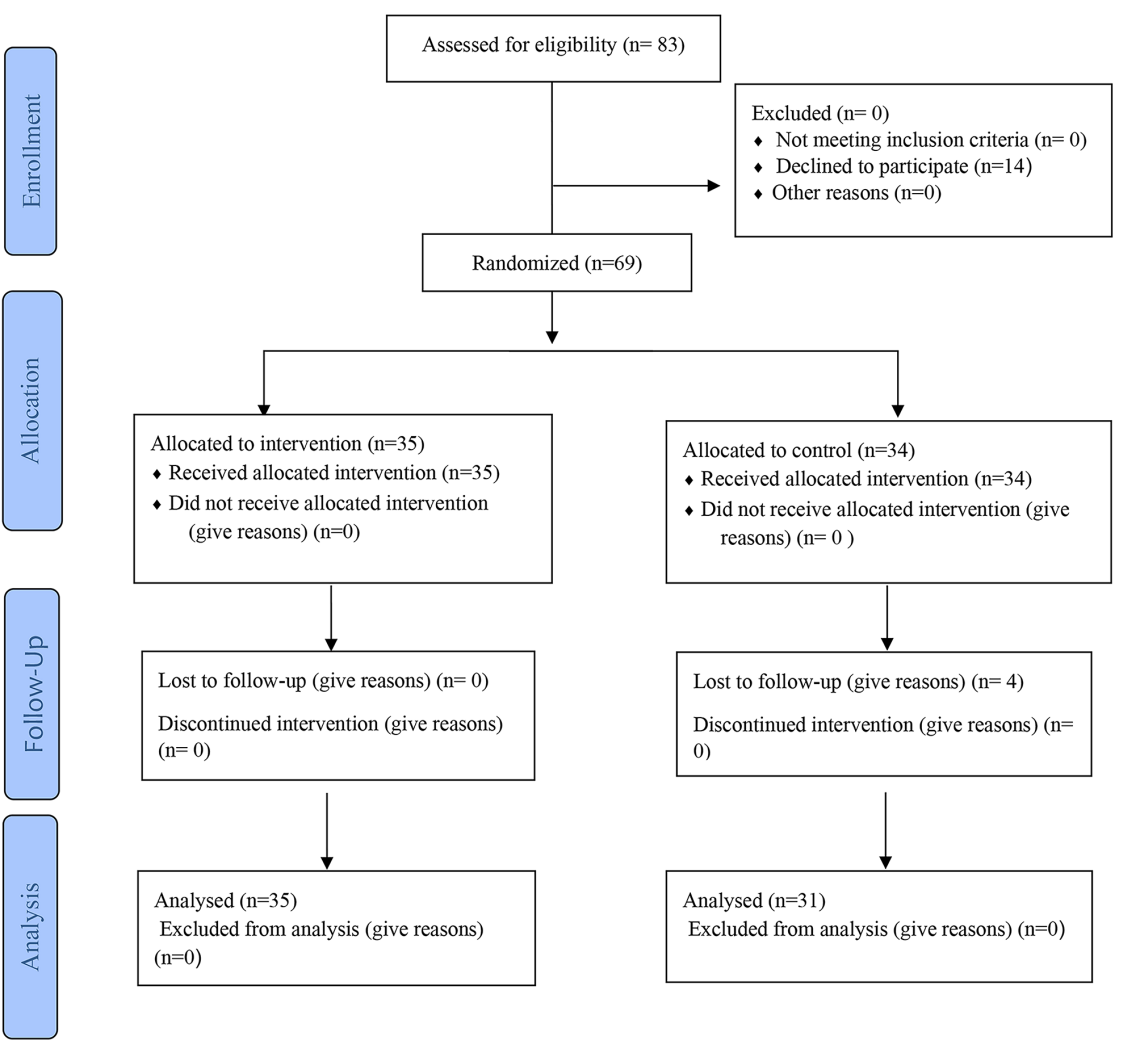
Flow diagram of the study, representing data collection points for the intervention group and the comparison group

The inclusion criteria included full-time nurse educators with academic ranks such as instructor and assistant professor who taught in the nursing education programs at baccalaureate, master, PhD degree levels. Educators who failed to complete the training program or the questionnaires for any reason were excluded. Finally, 65 nurse educators completed the questionnaires and four educators from the control group were excluded because of not completing questionnaires in the post-intervention phase (response rate = 94.20%) (Fig. 1).

### Measurement

To collect data, two tools were used. The first was demographic and professional information questionnaire consisting of nine items (see Table 1).

**Table 1:** Comparison of demographic and professional information between the two study groups

Variables	Categories	Intervention		Control		χ^2^	p- value
		n	%	n	%		
Gender	Male	6	17.1	6	20	0.08	0.76
	Female	29	82.9	24	80		
Marital status	Single	4	11.4	3	10	0.03	0.85
	Married	31	88.6	27	90		
Employment status	Committed	7	20	5	16.7		
	Contractual	10	28.6	12	40		
	Hired	18	51.4	13	43.3		
Degree	Ph.D.	19	54.3	16	53.3	0.006	0.93
	Master’s	16	45.7	14	46.7		
Residence in Kerman province	Yes	32	91.4	29	96.7	0.76	0.38
	No	3	8.6	1	3.3		
Location	Kerman	14	40	8	26.7	2.31	0.80
	Bam	5	14.3	6	20		
	Jiroft	5	14.3	5	16.7		
	Zarand	2	5.7	4	13.3		
	Sirjan	3	8.6	2	6.7		
	Rafsanjan	6	17.1	5	16.7		
Majors	Medical surgical nursing	13	37.1	10	33.3	5.53	0.85
	Psychiatric nursing	2	5.7	2	6.7		
	Community health nursing	4	11.4	3	10		
	Pediatric nursing	4	11.4	4	13.3		
	Critical care nursing	7	20	4	13.3		
	Nursing management	1	2.9	2	6.7		
	Others	4	11.5	5	16.7		
History of attendance in cultural competence training courses	None	29	82.8	23	76.7	1.12	0.77
	1 course	3	8.6	4	13.3		
	2 courses	1	2.9	2	6.7		
	≥ 3 courses	2	5.7	1	3.3		
Curriculum status in terms of providing culturally congruent care	Little	21	60	13	43.3	2.91	0.40
	Somewhat	4	11.4	8	26.7		
	Moderate	8	22.9	7	23.3		
	High	2	5.7	2	6.7		
		M ± SD		M ± SD		Independent t- test	P-value
Age		39.43 ± 7.92		39.70 ± 6.47		-0.15	0.88

The second was the Cultural Diversity Questionnaire for Nurse Educators (CDQNE) developed by Sealey to measure the cultural competence of nurse educators [24] .Then, the Cultural Diversity Questionnaire for Nurse Educators Revised (CDQNE-R) was revised by Yates [25]. The CDQNE-R has 41 items based on the concepts of Campinha- Bacote’s model of cultural competence. It measures cultural awareness (items 7, 10, 28, 31, 36, 37, 38, 40), cultural knowledge (items 5, 11, 14, 16, 17, 21, 22, 29, 32, 35, 39), cultural skills (items 1, 8, 9, 12, 18, 33, 34, 41), cultural encounters (items 3, 13, 15, 20, 23, 30), and cultural desire (items 2, 4, 6, 19, 24, 25, 26, 27). The CDQNE-R also includes a subscale that measures transcultural teaching behaviors (TTB), and 10 items (items 2, 9, 13, 16, 21, 24, 26, 28, 31, 33) are embedded within the other five subscales. The TTB subscale is related to practices of nurse educators in the classroom and the clinical settings and their commitment to teaching cultural knowledge and skills, and the degree to which they include transcultural nursing concepts in the educational courses. The responses were based on a five-point Likert scale) from 5 = strongly agree to 1 = strongly disagree). Scores for each subscale and total score were between 1 and 5. To interpret the responses, the researchers used Benner’s theory to categorize respondents into five levels: Cultural Novice (1–1.5), Culturally Advanced Beginner (1.6–2.5), Culturally Competent (2.6–3.5), Culturally Proficient (3.6–4.5), and Cultural Expert (4.6–5). The content validity of the CDQNE-R was confirmed by a panel of experts and a factor analysis. Its reliability was determined using internal consistency, and the Cronbach’s alpha coefficient was reported to be 0.63-82 for the subscales and 0.83 for the CDQNE-R scale [24, 26]. In order to use the CDQNE-R, the researchers translated it into Persian using the forward-backward translation method. The qualitative content validity of the CDQNE-R was corroborated by 10 nurse educators, and its reliability was determined through internal consistency method. The Cronbach’s alpha coefficient for the CDQNE-R was α = 0.94 and it was α = 0.67–0.88 for the subscales.

### Data collection

After obtaining the necessary permissions, the corresponding author prepared a list of all eligible nurse educators, including their names, telephone numbers, and email addresses. Then, she contacted and gave them the necessary information about the study objectives. Participants who volunteered to participate in the study were assigned into the intervention and control groups using a table of random numbers. The first researcher created two WhatsApp groups for the control and intervention groups, and then electronically provided the written consent form and the pre-test questionnaire to both groups. She also explained them how to complete the questionnaire and shared a guide for answering questions in both WhatsApp groups. Some participants wanted to receive the questionnaire link via email. The post-test data was collected electronically one month after the training program.

### Description of the training program

The educational content was prepared by conducting a review of the literature [2, 6, 7, 27–30] and two nursing faculty members confirmed its content validity (Table 2). To implement the training program, the researchers presented information about the process and schedule of the training program in WhatsApp groups. After the necessary coordination with the intervention group, the researchers trained transcultural care in three 2-hour sessions (two online sessions and one offline session) within a month. Online sessions were held through the Sky Room, which included lectures, questions and answers, PowerPoint, presentation of case reports and examples. For the offline session, the educational content recorded in the Sky Room was shared with the intervention group through WhatsApp. In addition, the intervention group was provided with recorded content of all sessions, several short educational videos, several up-to-date and related articles, and various educational self-learning texts on WhatsApp. The online sessions were held at 12–14 PM to minimize interference with the participants’ work schedule. The time of the training program and the link to join the class were posted on WhatsApp as well as in the news section of the Razi school of Nursing and Midwifery website one week before the training program. Moreover, the researchers closely monitored the study conditions to ensure that the intervention group completed the offline session. For this purpose, reminders were sent to them on WhatsApp and they were asked to give feedback to the researchers. All participants in intervention group confirmed that they studied the content of the offline session and completed the training program. The participants of the intervention group were asked to refrain from sharing information until the end of the study. The control group only received teacher empowerment programs and received a valid certificate for subject knowledge enhancement.

**Table 2:** Themes covered in the training curriculum

Sessions	Main topics	Contents
1(online)	-Introduction and orientation with concepts of cultural care and the importance of education -Integration of cultural care into the nursing process	- The need for multicultural education in higher education - Definition of culture and factors affecting cultural diversity - Cultural diversity and nursing education program - Cultural competencies and training program to prepare professors and students - Concerns and strategies in multicultural education Strategies for integrating cultural and linguistic competence into nursing education - National documents/standards - Healthy People 2020 etc. for culturally congruent care - The importance of diversity in health care as well as its challenges and opportunities - Communication skills and culture, and communicating between cultures about health and disease - Definition of cultural care, cultural competence - Prerequisites for cultural competence - The importance of cultural competence among nursing students and educators - Integration of cultural care into the steps of nursing process (assessment, nursing diagnoses, implementation of interventions, and evaluation) Assessment: LEARN model, BELIEF model, 4 c model and documentation of clients’ health beliefs/model of their illness and health - Cultural care and linguistic services standards - Identification of healing traditions and beliefs of clients at the workplace and education
2 (online)	-Models of cultural care and cultural competence -Organizational cultural competence	- Comparing and applying models of effective cultural care: Leininger’s cultural care model -Giger and Davidhizar’s evaluation model -Purnell’s model of cultural competence - Campinha – Bacote’s model of cultural competence in providing health care - Orlandi’s model -Patterns for health education programs The Cultural Assessment Framework Airhihenbuwa’ The PEN-3 cultural model - Practices related to cultural competencies in different environments - Organizational cultural competence and methods of creating cultural competence in the organization and its barriers - Strategies and barriers to organizational competency (clinical, organizational and structural) - Workforce with cultural competence - Case reports and examples
3 (offline)	-Barriers, outcomes and cultural competence -Providing cultural care to immigrants	- Bio-physiological determinants of health, and illness of minority groups - Barriers to effective cultural care, such as diagnostic inaccuracies, unintentional patient exploitation, racial and ethnic inequalities and disparities, group communication difficulties - Inequalities in health and socio-economic status - Equality of health, social justice, health literacy -Positive health outcomes related to cultural competence and cultural care - Cultural competence in providing services to immigrants -Immigration status and conditions and cultural diversity in immigrants - Complementary medicine and common diseases among immigrants - Process of acculturation in immigrants - Patterns of cultural acculturation: cultural assimilation, separation, integration, marginalization - Culturally congruent care for immigrants - Strategies for health education programs with cultural competence for immigrant clients - Summary and conclusions

### Statistical analysis

Data were analyzed using SPSS21 as well as descriptive (frequency, percentage, mean score, and standard deviation) and inferential (Chi-square, independent t-test, and paired t-test) statistics. The Kolmogorov-Smirnov test showed a normal data distribution. The significance level was considered at 0.05.

## Results

### Participants’ demographic and professional information

Table 1 showed that the mean ages of nurse educators in the intervention and control groups were 39.43 and 39.7 years, respectively. Most of the educators in the intervention and control groups were female (0.82, 80%), married (88.6, 90%), hired (51.4, 43.3%), held a PhD in nursing (54.3, 53.3%), and had a major in medical surgical nursing (37.1, 33.3%). They also worked in nursing school affiliated with the medical university of Kerman (40, 26.6%), and lived in Kerman province (91.4, 96.7%). The majority of educators in the intervention and control groups had not attended any cultural competence training courses (82.9, 76.7%) and believed that current curriculum provided little culturally congruent care for students (60, 43.3%). No significant difference in demographic and academic information was found between the intervention and control groups.

### Comparison of changes of cultural competence

Table 3 shows the level of the total cultural competence and scores for six CDQNE–R subscales in both groups before and after the training program. The between-group comparisons indicated no significant difference in the score of cultural competence between the intervention (3.29 ± 0.58) and control groups (3.24 ± 0.58) before training. According to categorization of CDQNE–R to interpret the responses based on Benner’s theory, both groups were culturally competent (t = 0.05, p = 0.95). The score of cultural competence in the intervention group (3.80 ± 0.7) significantly increased compared to the control group (3.23 ± 0.67) after training, so that the culturally competent participants became culturally proficient with a very large effect size (Cohen d = 0.83, t = -4.76, *p* = 0.001). According to the scoring of CDQNE–R based on Benner’s theory, scores 3.6–4.5 represented culturally proficient.

**Table 3:** Comparison of changes of the total cultural competence and scores of six CDQNE–R subscales between the control and intervention groups before and after the training program

Variables	Time	Pre-intervention	Post-intervention	Mean difference	ES^*^ (Cohen d)	Paired *t*-test	*P*- value
	Groups	M ± SD	M ± SD				
Cultural awareness	Intervention	3.75 ± 0.64	4.10 ± 0.67	0.35	0.53	-2.78	**0.009**
	Control	3.71 ± 0.60	3.75 ± 0.63	0.04	0.08	0.49	0.62
	Independent *t*- test	-0.03	2.39				
	*P*-value	0.97	**0.02**				
	ES^*^ (Cohen d)	0.01	0.59				
Cultural knowledge	Intervention	3.02 ± 0.67	3.65 ± 0.81	0.63	0.84	-4.44	**0.001**
	Control	2.88 ± 0.71	2.97 ± 0.80	0.08	0.11	-0.72	0.47
	Independent *t*- test	0.80	3.38				
	*P*-value	0.42	**0.001**				
	ES^*^ (Cohen d)	0.20	0.84				
Cultural skills	Intervention	3.22 ± 0.65	3.84 ± 0.66	0.62	0.94	-4.81	**0.001**
	Control	3.23 ± 0.68	3.05 ± 0.78	0.18	0.24	1.33	0.19
	Independent *t*- test	-0.05	4.41				
	P-value	0.96	**0.001**				
	ES^*^ (Cohen d)	0.01	1.09				
Cultural encounters	Intervention	2.88 ± 0.81	3.40 ± 0.88	0.52	0.61	-3.36	**0.001**
	Control	2.93 ± 0.74	2.82 ± 0.86	-9.88	0.13	0.84	0.40
	Independent *t*- test	-0.24	2.68				
	*P*-value	0.80	**0.009**				
	ES^*^ (Cohen d)	0.06	0.66				
Cultural desire	Intervention	3.55 ± 0.53	3.93 ± 0.74	0.38	0.59	-3.24	**0.003**
	Control	3.58 ± 0.54	3.55 ± 0.69	0.03	0.04	0.35	0.72
	Independent *t*- test	-0.28	2.13				
	*P*-value	0.78	0.03				
	ES^*^ (Cohen d)	0.05	0.53				
Transcultural teaching behaviors	Intervention	3.34 ± 0.72	3.89 ± 0.74	0.55	0.75	-4.03	**0.001**
	Control	3.34 ± 0.70	3.27 ± 0.79	0.07	0.09	0.59	0.55
	Independent *t*- test	0.04	3.23				
	P-value	0.96	**0.002**				
	ES* (Cohen d)	0	0.81				
Total of cultural Competence	Intervention	3.29 ± 0.58	3.80 ± 0.7	0.51	0.79	-4.21	**0.001**
	Control	3.24 ± 0.59	3.23 ± 0.67	-0.01	0.09	0.61	0.54
	Independent *t*- test	0.05	-4.76				
	P-value	0.95	**0.001**				
	ES* (Cohen d)	0	0.83				

Bold p-values are significant at level of ≤ 0.05.

Effect size (ES): 0-0.2 = small effect, 0.2–0.5 = moderate effect, > 0.5–0.7 = large effect, and > 0.7 = very large effect

Moreover, the intervention group had higher scores in all subscales of CDQNE-R than the control group, so that the effect sizes of subscales in the intervention group were between 0.53 and 1.09 after training.

The within-group comparisons of the intervention group showed that the total scores of CDQNE-R and its subscales improved after training compared with before. According to the scoring of CDQNE–R based on Benner’s theory, the intervention group promoted from being culturally competent to culturally proficient.

The mean differences showed that training program had the highest effect on the cultural knowledge subscale (M diff = 0.63) and the lowest effect on cultural awareness subscale (M diff = 0.35).

In addition, the multivariate linear regression (Forward stepwise method) was conducted to eliminate the impact of confounding variables on the cultural competence (such as age, gender, marital status, employment status, degree, residence in Kerman province, location, majors, history of attendance in cultural competence training courses, and curriculum status in terms of providing culturally congruent care). The results showed that these variables were not significant predictors of cultural competence among nurse educators in this study (Table 4).

**Table 4:** Multivariate regression model for demographic and professional information variables

	Unstandardized coefficients		standardized coefficients		
	β	Standard error	β	t	p-value
Constant	4.42	1.05		4.20	**0.001**
age	-0.2	0.01	-0.27	-2.01	0.51
Gender	-0.36	0.21	-0.18	-1.67	0.1
Marital status	0.34	0.23	0.14	1.45	0.15
Employment status	0.13	0.08	0.22	1.67	0.1
Degree	0.09	0.18	0.06	0.5	0.61
Residence in Kerman province	-0.26	0.35	-0.08	-0.72	0.47
Location	0.05	0.04	0.13	1.22	0.22
Majors	-0.02	0.03	-0.09	-0.78	0.43
History of attendance in cultural competence training courses	0.13	0.09	0.17	1.49	0.14
Curriculum status in terms of providing culturally congruent care	0.13	0.09	0.18	1.52	0.13
Time	3.01	1.30	0.01	0.39	0.21
group	-0.61	0.15	-0.41	-3.91	**0.001**

Bold p-values are significant at level of ≤ 0.05

## Discussion

The present study evaluated the effect of a virtual training program on the nurse educators’ cultural competence. According to the results, the training program significantly increased cultural competence and all its dimensions in the intervention group compared with the control group. According to the scoring of questionnaire based on Benner’s theory, cultural competence of intervention group was promoted from culturally competent to culturally proficient level. These results were consistent with previous studies that confirmed the effectiveness of training programs in enhancing the cultural competence of nurses and nursing students [30–38]. However, our search revealed that only one study investigated the effectiveness of such a program for nurse educators. The results showed that most of the nurse educators only had cultural awareness before the intervention, but their cultural competence increased in all areas after the intervention. In addition, the nurse educators acknowledged their role in strengthening and improving students’ cultural competence and reported an increase in their self-efficacy [20]. While interventional studies on the competence of nurse educators are limited, descriptive studies investigating nursing programs and the cultural competence of nurse educators were considered in this research. These studies provided valuable insights and suggestions for cultural competence training in nurse educators. For instance, Farber (2019) showed that nurse educators had little confidence in their cultural knowledge due to lack of international experiences, formal training, and educational workshops related to cultural competence [13]. Haller et al. (2018) reported a positive relationship between nurse educators’ cultural competence and their interaction with culturally diverse students. In addition, cultural knowledge was a strong predictor of the nurse educators’ cultural competence [17]. Abou Hashish et al. (2020) found that the majority of nurse educators had a moderate level of cultural competence and that high work experience, language skills, and participation in in-service cultural competence training courses increased their cultural competence [14]. Burns (2020) showed a moderate level of cultural competence among nurse educators and identified individual, professional nursing characteristics such as mental health expertise, continuing education, and proficiency in another language as predictors of cultural competence [39]. Chen et al. (2020) reported that faculty members had a moderate level of cultural awareness and competence and believed that collaboration between nurse educators and clinicians was an effective way to improve students’ cultural competences [40]. Baghdadi and Ismaile (2018) showed a moderate level of cultural competence among nurse educators, with cultural knowledge receiving the highest score and cultural skills receiving the lowest score. The researchers suggested that cultural competence training should be mandatory, and both theoretical and practical training should be provided to prepare nurse educators to work with diverse populations [12]. Furthermore, annual accreditation should be implemented to ensure ongoing professional development. However, one study reported that nurse educators were culturally competent in all dimensions and suggested that they continue to increase their cultural competence through activities such as reading or conducting research [41]. Walden (2020) conducted a qualitative study exploring nursing faculty members’ experiences in meeting the comprehensive needs of baccalaureate nursing students from culturally diverse backgrounds. The study revealed that faculty members could gain insight into cultural awareness, sensitivity, and competence by reflecting on their experiences, which could lead to a learning environment that meets the needs of all students. Furthermore, some faculty members lack the preparation needed to teach cultural content to culturally diverse student populations. Data analysis of qualitative studies on nursing education revealed five main themes: “faculty challenges, limited preparedness or lack of training, biases and assumptions, alternative strategies, and curricular and administrative significance.” By understanding nursing faculty’s teaching practices, they can go beyond routine processes of teaching and evaluate cultural competence experiences. This can lead to the identification of underlying assumptions and biases, allowing for the creation of a learning environment that accommodates all students, including those from culturally diverse backgrounds [6]. Other researchers investigated the factors affecting the implementation of cultural competence content in nursing education from nurse educators’ viewpoints. They reported the need for greater transparency and collaboration between nurse educators and faculty managers to ensure the inclusion of cultural competence in nursing education. In addition, achieving culturally competent nursing students require the commitment of faculty members and the transformation of nursing schools into cultural organizations [42]. Momeni et al. (2008) conducted a quantitative documentary analysis and examined the curricula of 26 nursing schools in Sweden. They showed that only 15% of the curricula included the concept of culture and only three faculties provided students with cultural competence education, and nurse educators did not prepare nursing students to work in a multicultural society [43].

Benner’s theory provides a useful guideline for instructors to design their curriculum in a more effective manner for both students and graduated practitioners. According to this theory, nursing practitioners can expand their competences through actual experiences. The model suggests that the progression from the novice step to the higher levels occur over time and with the accumulation of more experiences. However, it should be noted that simulators are valuable training tools that can be employed in the novice and advanced novice steps for providing nurses with required competences. The theory also highlights that advancing from the advanced novice to the component step is mainly a result of increasing self–confidence in practitioners, which stems from encountering various clinical conditions and the emergence of the feeling that they can rely on their skills. According to Benner’s theory, nurse educators can use simulators to facilitate the progress of nursing students in gaining clinical competences. Simulators come in various forms, including written simulations, simulated patients, role playing, maquettes. It is important to note that simulators are powerful tools that can facilitate the educational process in complex situations [44].

Creating culturally diverse environments among students, educators, and managers in universities can encourage students engage with and reflect on cultural differences. In addition, nurse educators’ experiences in the educational environment, their understanding of cultural competence, and their integration of cultural competence into the curriculum significantly improve the cultural competence of both educators and students. It is crucial that educators adopt a comprehensive approach to cultural competence in nursing programs that extends beyond classroom training. For example, they should organize training courses or workshops with clear learning objectives and create evaluation criteria for ranking educators [42]. Moreover, nurse educators can integrate various methods to educate cultural competence, including lectures, in-depth, interactive exercises and discussions, case study analysis, genograms, presentation of articles, selected readings and web-based learning and data collection, videos, simulations, role-playing, seminars, in-service- based learning, poster presentations, interview with clients, and development of a measuring tool [45].

The above studies have highlighted the need for nursing schools to train nursing students in the care of culturally diverse patients. Therefore, nurse educators are expected to act as role models for nursing students and possess a comprehensive understanding of their behaviors, actions, and attitudes that affect the development of cultural competence, and professionalism of nursing students. Moreover, cultural competence has not been integrated into nursing curricula. Nurse educators should revise nursing curricula to identify how to integrate cultural content into curricula and develop clear guidelines and standards. They can increase their own cultural competence, as well as that of other educators and students by using quantitative and qualitative research, evidence-based teaching methods, learning skills in different languages, creating culturally diverse spaces in universities, increasing transcultural communication between educators and students, developing transcultural care lesson plans, participating in international online and offline transcultural training and continuing education, experiential learning, scholarships, holding congresses and seminars in cultural care, immersive experience, and community-based services.

### Strengths and limitation

This study can be considered as the first national study in Iran to evaluate the cultural competence of nurse educators and the effectiveness a training program designed to enhance their cultural competence. The study provides insight into the cultural competence level of nurse educators and offers suggestions for nursing education to promote cultural competence. In addition, our experiences in implementing the virtual training can be beneficial for developing future programs aimed at fostering cultural competence of the nurse educators in nursing schools. The virtual training program (offline and online combination) was well-received by busy nurse educators who had various tasks to attend to.

Despite the valuable insights gained from this study, several limitations must be addressed. The results of this study were limited to nursing schools affiliated with medical universities of Kerman province in southeastern Iran. Moreover, the effectiveness of the training program was evaluated based on a self-assessment tool and one month after the training program. This might not give a real picture of nurse educators’ cultural competence. Future studies are needed with larger sample sizes and longer follow-up periods to assess the generalizability of findings to academic nurse educators in different settings.

## Conclusions

The results showed that the virtual training program could significantly improve academic nurse educators’ cultural competence and its dimensions. Nurse educators have the potential to affect cultural competence process in nursing students. They act as teachers, role models, and providers of culturally congruent care. Therefore, it is recommended to continuously develop and evaluate cultural competence among educators and students in universities through training programs. Professional cultural development programs for nurse educators should be compulsory and cover the key elements of cultural competence such as how to deal with problems when working with clients from multicultural backgrounds. The training programs should be conducted for new nurse educators, and their cultural competence should be evaluated annually. Qualitative studies should address factors affecting cultural competence from the viewpoints of nurse educators and find the best way to teach and learn cultural competence in nursing programs. In addition, further studies should compare cultural competence between nurse educators and their students to see how the level of cultural competence of the educators affects the level of cultural competence of the students.

## Data Availability

The data are available upon request to the corresponding author after signing appropriate documents in line with ethical application and the decision of the Ethics Committee.

## Abbreviations

CCT: Theory of Culture Care
CDQNE: Cultural Diversity Questionnaire for Nurse Educators
CDQNE: Cultural Diversity Questionnaire for Nurse Educators Revised
RN: Registered nurses
